# Formation Mechanism of Inter-Crosslink in DNA by Nitrogen Oxides Pollutants through A Diazonium Intermediate

**DOI:** 10.3390/ijms231810621

**Published:** 2022-09-13

**Authors:** Noemi Hernandez-Haro, Christian Solis-Calero, Rodrigo Casasnovas, Christophe Morell, Andre Grand, Juan Frau, Joaquín Ortega-Castro

**Affiliations:** 1 Departament de Química, Universitat de les Illes Balears, 07122 Palma de Mallorca, Spain; 2 Faculty of Pharmacy and Biochemistry, Universidad Nacional Mayor de San Marcos, Lima 15001, Peru; 3 Institut d’Investigació Sanitària Illes Balears (IdISBa), 07020 Palma de Mallorca, Spain; 4 Institut des Sciences Analytiques, Université de Lyon, Université Claude Bernard Lyon 1, UMR CNRS 5280, CEDEX, 69622 Villeurbanne, France; 5 Université Grenoble Alpes, CEA, CNRS, INAC-SyMMES, 38000 Grenoble, France

**Keywords:** DNA, inter-crosslink, pollutant, nitrogen oxide, DFT, MD

## Abstract

Outdoor air pollution is a mixture of multiple atmospheric pollutants, among which nitrogen oxide (NOx) stands out due to its association with several diseases. NOx reactivity can conduct to DNA damage as severe as interstrand crosslinks (ICL) formation, that in turn is able to block DNA replication and transcription. Experimental studies have suggested that the ICL formation due to NOx is realized through a diazonium intermediate (DI). In this work, we have modeled the DI structure, including a DNA double-strand composed of two base pairs GC/CG, being diazotized as one of the guanine nucleotides. The structural stability of DNA with DI lesion was essayed through 500 ns molecular dynamics simulations. It was found that the DNA structure of the oligonucleotide is stable when the DI is present since the loss of a Guanine–Cytosine hydrogen bond is replaced by the presence of two cation-π interactions. Additionally, we have studied the mechanism of formation of a crosslink between the two guanine nucleobases from the modeled DI by carrying out DFT calculations at the M06-L/DNP+ level of theory. Our results show that the mechanism is thermodynamically favored by a strong stabilization of the ICL product, and the process is kinetically viable since its limiting stage is accessible.

## 1. Introduction

Outdoor air pollution is a mixture of multiple pollutants originating from natural and anthropogenic sources. Transportation, power generation, industrial activity, biomass burning, and domestic heating and cocking are the predominant anthropogenic sources in many locations [1]. Europe’s most problematic pollutants in terms of harm to human health are PM (particulate matter), O_3_, nitrogen oxides (NO_x_), and SO_2_. In Europe, about one-third of the PM_10_ concentration and half of the PM_2.5_ concentration measured consist of inorganic chemical substances, such as ammonium (NH_4_^+^), nitrate (NO_3_^−^), and sulfate (SO_4_^2−^) ions. O_3_ is a secondary pollutant formed in the troposphere. Its formation comes from the chemical interaction of NO_x_ and volatile organic compounds (VOC) precursor gases. NO_x_ is emitted during fuel combustion, and high concentrations are associated with swelling processes and liver, blood, and spleen problems. Data show that 5% of the population suffers excessive exposure to these gases [2,3].

The integrity of the genome may be seriously affected due to DNA damage by various substances, exogenous and endogenous. Pollution, radiation, and alkylating agents are mostly reasons for the exogenous factors that damage the DNA deeply. The most important carcinogenic substances that affect humans are gases in the air [4]. Reactive Oxygen species (ROS), Reactive Nitrogen species (RNS), and alkylating agents (RNOx) are common and drastically damage the structures and functions of the DNA by a great diversity of mechanisms, such as modifications to the nitrogenous bases [5,6]; the oxidation of the sugar moiety [7,8]; DNA single and double strand breaks [9]; the alkylation of one or both DNA chains by bifunctional agents that produces intra- or inter-crosslink DNA (ICL) and inhibits the transcription and replication processes [10], protein–DNA crosslinking. These processes can lead to severe consequences; mutations due to miscoding or modifications of DNA are the leading cause of carcinogenesis [11], aging, neurodegenerative diseases, arthritis, and AIDS [12,13,14,15,16]. The DNA damages are mainly modifications in the 2′-deoxyribose or the nucleobase of one nucleotide. These modifications can be changes in the structure and chemistry properties that could cause the loss of chemical functions. There is strong experimental evidence indicating that guanine is the most probable site for hole trapping, creating electron holes that migrate along the DNA stacks [9,17,18,19]. Since GG- and GGG-stacked guanine sequences have lower ionization potential than single guanine, such sequences are even more probable sites for undergoing the oxidation process [20,21,22,23]. If these kinds of lesions were not repaired, they could produce mutations. For example, the damage caused by these species generates products of deamination leading to the conversion of C to U, G to xanthine, G to oxanosine, 5-methylcytosine to T, A to hypoxanthine, and inter- or intra-crosslink too [24,25]. The type and number of lesions depend on the concentration of different species (CO_2_, HCO_3_^−^, NO_2_^−^, CO_3_^2−^). One of the lesions that is more dangerous and more difficult to repair is the inter-crosslink formation. It has been estimated that a single unrepaired ICL can kill a bacterial or yeast cell, while about 40 ICLs can kill a repair-deficient mammalian cell [26].

The DNA damaging effects of nitrous acid have been studied in the past, and their involvement in the formation of ICL has been observed [24,27,28,29]. Both the ICL and ICL-protein can be formed by NO^−^ derivatives [30,31]. In the DNA double helix structure, this kind of ICL shows a preference for the formation in the sequence dCpG. Early works are traced to Shapiro et al. [32] to propose candidate structures for the crosslinks and to determine these structures using NMR and MS tentatively. Two different structures were found, i.e., a dG–dG crosslink and a dG–dA crosslink. Subsequent work has shown the dG–dG structure to be highly important [32]. Further studies by Kirchner et al. [33] have shown the formation of the dG–dG interstrand crosslink by reacting nitrous acid with oligomers. It also seems that the formation mechanism of the ICL occurs through diazotization [28,29]. The ONOO^-^ mainly reacts with dG in the DNA, forming a wide variety of modifications known as 8-nitrodG and 8-oxodG. The damage caused by these species may be the products of a deamination reaction (guanine to xanthine or oxanosine), besides inter- or intra-crosslinks [24,25]. The crosslink may interfere with DNA synthesis or transcription due to the inability of the strands to dissociate.

A better knowledge of the formation mechanism of the ICL lesions is necessary. For example, how the DNA sequence influences their formation and the changes that occur once these ICLs are created. This knowledge could lead to effective strategies for treating various diseases such as cancer and the design of drugs that repair ICLs. To contribute more to the knowledge of this type of success, a computational approach has been used to study the mechanisms that describe the crosslink formation by RNS.

## 2. Results and Discussion

Living organisms are continually exposed to many DNA-damaging agents that can affect health and modulate disease states. It is known that DNA is an intrinsically reactive molecule and is highly susceptible to chemical modifications by endogenous and exogenous agents [34]. Cells respond to DNA damage with their own mechanisms that can repair them: base excision repair (BER), nucleotide excision repair (NER), mismatch repair (MMR), homologous recombination (HR), and non-homologous end joining (NHEJ) remove a lesion from one strand of the duplex. An ICL that covalently binds both strands of a duplex DNA requires a more elaborate repair mechanism. To understand the mechanism involved in the recognition and repair of ICLs, the DNA structure containing such a lesion must be known. The formation of this interstrand crosslink has been of considerable interest; however, because of the low levels of crosslinked DNA, the resulting detection and quantitation are difficult.

The ICL formation process in the double-helix structure of DNA is a complex process that starts with the formation of one lesion in the DNA. These lesions may be formed in the major and minor grooves of DNA, intercalated between two base pairs or connecting two opposing bases through atoms involved in base pairing. Substances with reactive nitrogen atoms, such as nitrous acid, are typical in outdoor air pollution and can cause sequence-specific DNA damage that initiates and promotes diseases like cancer [4,35]. Specifically, nitrous acid can produce crosslinks in duplex DNA by the diazotization of an exocyclic amino group of a guanine residue, followed by displacement by a nucleophile, which may be the amino group of another DNA residue [28]. Then, the first aim of this work was to develop a model of a DNA double strand that could be used as a starting point for obtaining a mechanism of the reaction of the formation of ICLs.

**Figure 1:**
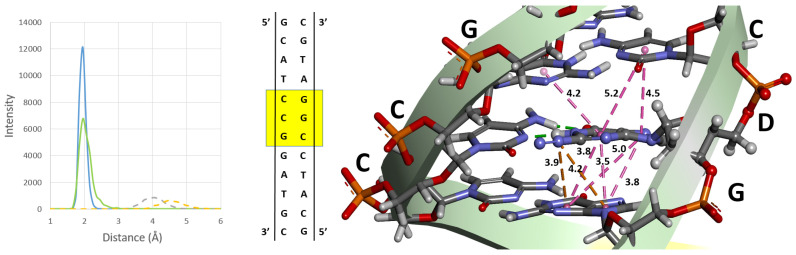
Histograms of distances during the MD simulations (Left Panel) and a representation of principal interaction schemes in the dodecamer DNA models for the DI (Central and Right Panel). The solid blue and green lines represent the histogram of hydrogen bonding for the G–D pairs in the diazonium DNA model and the dashed gray and yellow lines are the major histograms for the cation-π interactions during the MD simulations. A representative figures of the base in the oligonucleotide model (Right Panel) and its place in the dodecamer (yellow square) are also shown. Pink lines are pi–pi stacked interactions; orange lines are cation–π interactions.

A model of a DNA double-strand including two cytosine and two guanine nucleotides, with one diazotized guanine, was built (Figure 1). This model was based on previous experimental studies that measured the preference for crossovers in the nucleotide sequence 5’-CCGG with nitrous acid [28,33]. It has been proposed that this diazonium intermediate (DI) is formed by the reaction of an exocyclic amine group of a guanine nucleotide and HNO_2_ or NO [36,37,38]. In the model, similar nucleotides were in opposing strands. This structural disposition was chosen because the formation of ICLs is conducted by reacting with a hydrated form of nitric oxide when adjacent guanine residues are situated in opposing strands [39]. This model was also used for analyzing, via molecular dynamics simulations, the variety of structural distortions in DNA due to the formation of this DNA double-strand diazonium ion intermediate.

**Figure 2:**
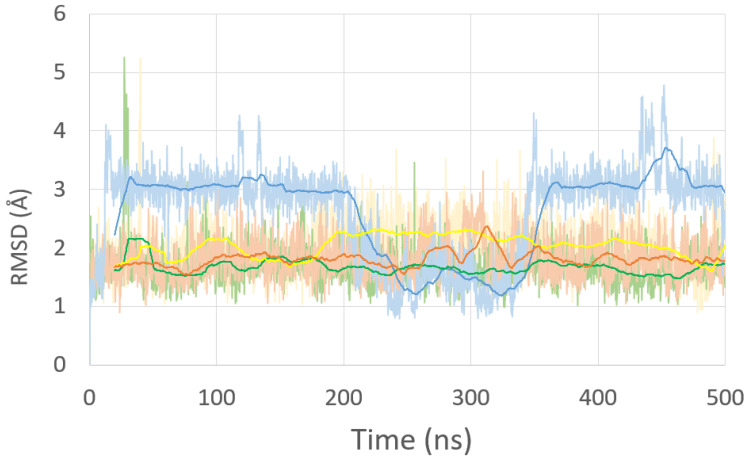
Root mean square deviation (RMSD) of the residues of the native (green), diazonium models (yellow), ICL-A model (brown), and ICL-B model (blue) during 500 ns of MD simulation.

Diazonium intermediates could induce a variety of structural distortions in DNA. The study of the changes suffered by the inclusion of the modification was carried out theoretically through 500 ns-long molecular dynamics trajectories of the native and the DI structures. Overall, the diazonium model remained stable and close to the native structure throughout the simulation (Figure 2), so this intermediate causes no denaturation of the DNA structure within the simulated timescale. The helical parameters were measured to characterize the secondary structures of the nucleic acids with the Web-3DNA and CURVES+ online servers [40,41]. The averaged base step parameters are shown in Tables S1 and S2. As can be seen in these tables, there are few differences between both structures within the fluctuation range. Only a slight increase is detected in the Shift, Slide, Rise, and Tilt where the lesion is found. Figure 1 shows a representation of the principal interaction of the DI with the nucleobases around it. The diazonium inclusion in the minor groove exhibits the disappearance of one of the three hydrogen bonds of the D–C base pair and the appearance of two cation–π interactions between the positively charged nitrogen of diazonium and the pi cloud of the stacked Guanine. These interactions are strong enough to stabilize the modified DNA during the simulation. It is estimated that base stacking contributes as much as half of the total stabilizing free energy of a base pair in duplex DNA [42].

**Scheme 1:**
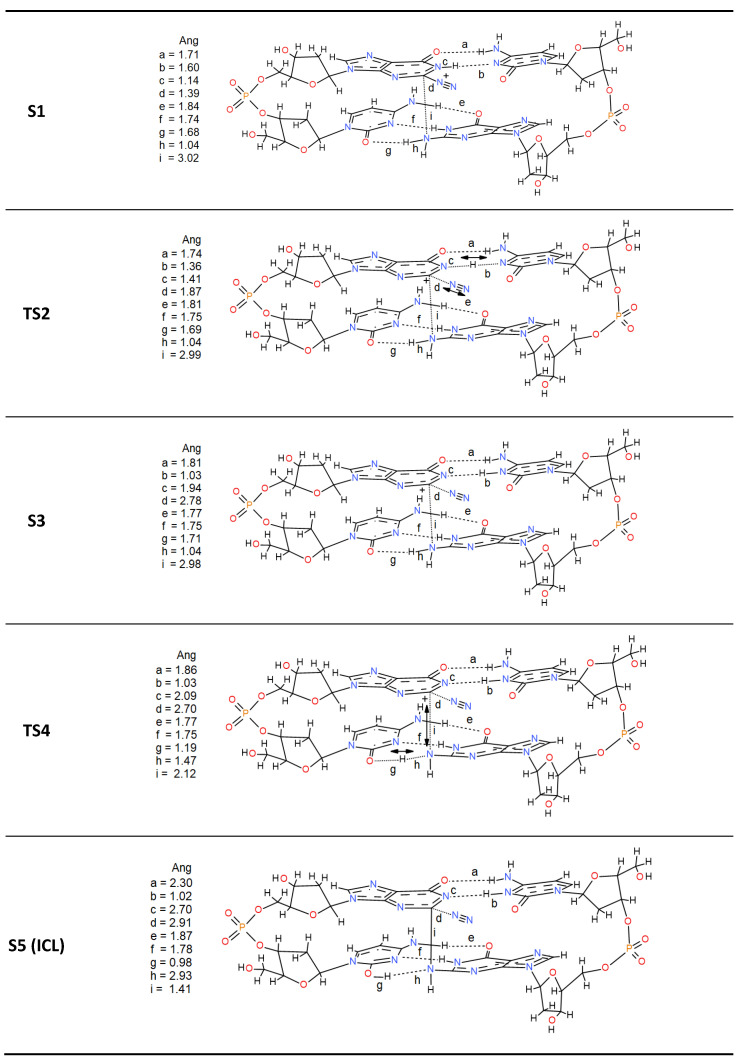
Mechanism of formation of Guanine-Guanine ICL (S5) from the diazonium adduct (S1).

**Figure 3:**
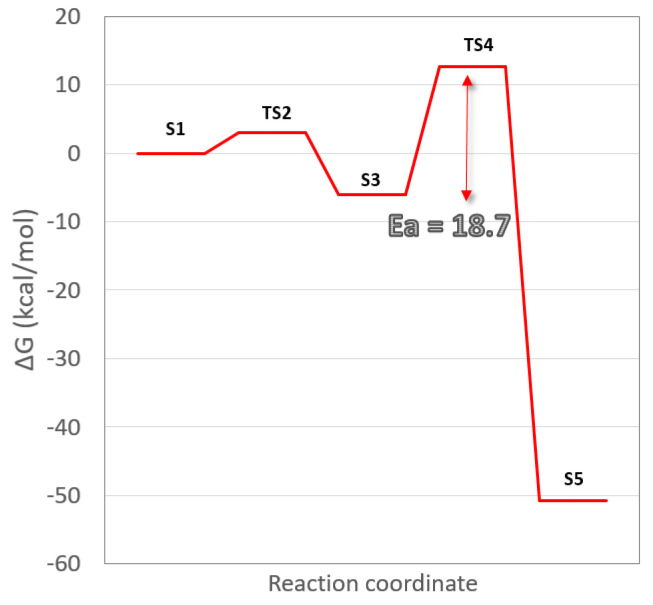
Free energy (ΔG) profile for the formation of Guanine–Guanine intercross link.

The work’s main objective is to know the reaction mechanism that yields the ICL as a final product. A representative frame of the diazonium-modified DNA simulation was chosen to achieve this. Then, DFT calculations were used to study the most reasonable steps to achieve that final product. The chosen model contains four complete nucleotides, where, in addition to the lesion and the cytosine that forms its natural base pair, the second pair of stacked nucleotides C-G is added (Scheme 1). The reaction starts with a concerted step by a hydrogen transfer of the imine of the diazonium intermediate (S1) to its cytosine counterpart and the simultaneous release of the diazonium group (nitrogen molecule output). The reaction occurs via a transition state TS2, where these changes are indicated with arrows in Scheme 1. TS2 shows how the iminic hydrogen is halfway between the lesion and the cytosine (1.36 vs. 1.41 Å) and how the N_2_ molecule leaves through the plane formed by the base pair. The diazonium charge is then transferred to the aromatic structure. This restructuring is kinetically viable since the energy barrier for this step is only 3.1 kcal·mol^−1^ (Figure 3). The formed N_2_ is conserved in the subsequent steps without intervening in the process, keeping a distance that is never less than 2.70 Å. With this first concerted step, a stabilization of 6.0 kcal·mol^-1^ from the reactant state occurs (S1). This step agrees with the thermodynamic study carried out by Glaser et al. [43], who predicted a value of 8.7 kcal·mol^−1^ with the RHF/B3LYP/6-31G* methodology. The last step of the mechanism is the formation of the ICL. The process is carried out through TS4 also in a concerted way. TS4 shows the bond formation (1.41 Å) between Guanines through the nucleophilic attack of the amine nitrogen to the carbocation of the lesion. In this step of the reaction, there is also the transfer of hydrogen between the attacking amine of the stacked Guanine and its base pair producing the enol form of cytosine. This process is the limiting step of the reaction with an activation energy of 18.7 kcal·mol^−1^. However, a strong stabilization is produced (−50.8 kcal·mol^−1^ from the reactants) in S5, which determines the orientation of the reaction. As can be seen, the stabilization of the final product is very high, causing the equilibrium to shift towards the formation of the ICL. This mechanism shows that the inter-crosslink formation process is thermodynamically favored.

Finally, two additional models were built with the same DNA sequence previously used to visualize the changes a DNA fragment undergoes, including an inter-crosslink of this type. The first model (ICL-A) is the final product of our reaction mechanism (S5, Scheme 1). The second model (ICL-B), originally synthesized by Shapiro et al. [32] as the final product of the process, preserves both iminic nitrogens within the structure. In both models, the inclusion of the ICL forces the cytosines that were previously pairs of both guanines to rotate. However, great differences are found when inspecting the MD simulation trajectories (Figure 2). While the ICL-A model (brown line) shows a deviation similar to the rest of the previous dynamics, the ICL-B model (blue line) suffers a strong deviation in the ranges of 15–200 ns and 350–500 ns.

**Figure 4:**
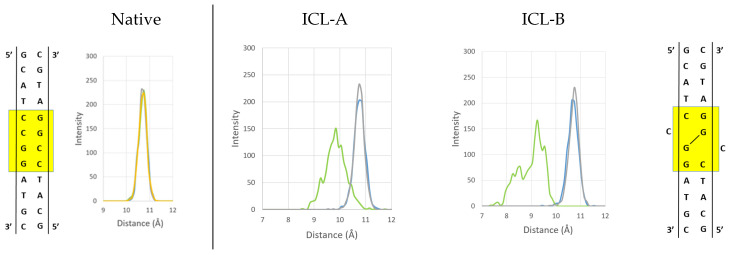
Histograms of distances between the C1’ of the attached sugars for each base pair of the central GGCC sequence (yellow square) of the native structure and the G-ICL-G sequences of the ICL-A and ICL-B models presented during the MD simulations.

An in-depth analysis focuses on the behavior of the ICL itself within the oligonucleotide. While for the ICL-A model, the cytosine bases adjacent to the lesion are flipped out of the helix and generally reside in the minor grove, in the ICL-B model, this behavior does not occur when the cytosines are soaked in the solvent longer than they are placed in the minor groove. Figure 4 presents the radial distribution function between the C1’ of the attached sugars for each base pair of the central GGCC sequence of the native structure and the G-ICL-G sequences of the ICL models. In all cases, the base pairs show a uniform distance peak of around 10.7 Å, and a shortening of the distance between the C1’s is only observed for the ICLs. However, the distance between their sugars (9.9 Å) is closer to the ideal for the ICL-A model than that found in the ICL-B model, which presents a maximum at 9.2 Å with greater dispersion of values (7.45–10 Å).

**Figure 5:**
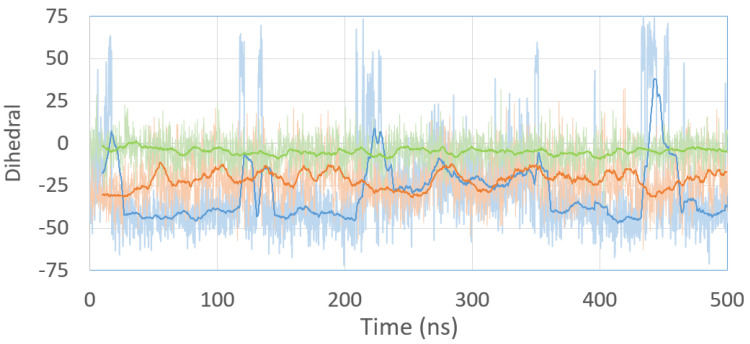
Propeller twist angle between Guanine analogs of the ICL-A (orange) and ICL-B (blue) models and the propeller twist angle of the G-G base pair of the native (green) model during 500 ns of MD simulation. The solid line represents the moving average of each simulation.

**Figure 6:**
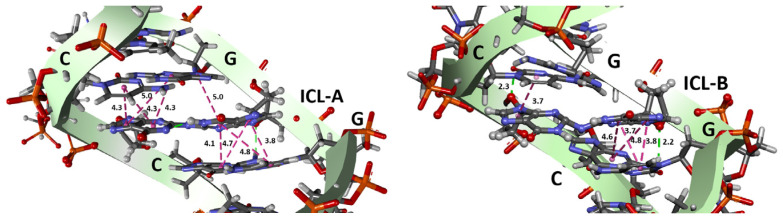
Representative structures of the ICL interactions (Å) with the base pair stacking in the oligonucleotide models are shown. The pink lines are pi–pi stacked interactions, and the green lines are hydrogen bonds.

For the ICL-A model, the crosslinked guanines have a stable propeller twisting (Figure 5, orange line) during the entire MD simulation. This allows the ICL to stack well on the adjacent base pairs and helps cytosine to stay in the minor groove. However, the ICL-B model presents more significant fluctuations (Figure 6, blue line), which indicates the reduced stability of the system. From 200 to 350 ns, both propeller twister angles are comparable, coinciding with the only zone in which model B presents the cytosine aligned in the minor groove. This different behavior is due to the presence of iminic hydrogen in both guanine analogs in ICL-B, while the absence of one of them in the ICL-A model results in the greater structural stability of the lesion within the oligonucleotide. These results agree with those obtained experimentally from NMR spectroscopy [29], where the iminic proton could not be detected. Representative structures of both models can be seen in Figure 6.

## 3. Materials and Methods

All the molecular dynamics (MD) simulations were performed with GROMACS 2016.4 software, Groningen, Netherlads [44,45,46,47] with the GROMOS96 53A6 force field [48]. All systems were minimized using the steepest descent algorithm and then went through a 100-ps NVT equilibration, a 100-ps NPT equilibration, and a 500-ns NPT production run. The leap-frog integrator with a 2 fs time step was used throughout. The DNA atoms were position-restrained during the equilibration and no restraints were used during the minimization and production. The temperature was kept at 300 K with a velocity rescale thermostat [49] and the pressure at 1 bar with the Parrinello–Rahman barostat [50]. The τ_T_ for the thermostat was set to 0.1 ps during the equilibration and production phases. The τ_P_ for the barostat was set to 2.0 ps during both the NPT equilibration and the production. Both nonbonded cutoffs (van der Waals and short-range electrostatics) were set to 1.0 nm. Long-range electrostatics was treated with the PME method [51] with a 0.16 nm grid spacing during the equilibration and production. Both energy and pressure dispersion corrections were applied. Periodic boundary conditions and the minimum image convention were used. Snapshots were collected every 2 ps. The crystal structure of the DNA was chosen according to the synthesis and characterization experiments previously carried out by Edfeldt et al. [28] and Kirchner et al. [33]. DNA models were solvated using a molecular water box with equilibrated water (TIP3P) with sodium ions to neutralize the system’s total charge. The trajectories were analyzed to obtain: (1) the root mean square deviation (RMSD) concerning the initial structure; (2) the radial distribution functions (3) the distances between the coupled bases; (4) the analysis of intra or intermolecular hydrogen bond distances, and (5) the propeller twist angle (more details of the statistical analysis can be seen in the Support).

The reaction mechanism of ICL formation was carried out, starting from a frame of the diazonium structure obtained from the MD simulations. All the calculations were performed in the framework of the density functional theory (DFT) with the DMol3 package of Accelrys, Inc., San Diego, CA, USA [52,53,54] using the double numerical basis set with augmented diffuse valence orbitals on heavy atoms and polarization functions on all atoms (DNP+) [52] and the Minnesota 2006 local functional (M06-L) local exchange-correlation function [55]. The maximum number of numerical integration mesh points available in DMol3 was chosen for our computations, and the threshold of the density matrix convergence was set to 10^−5^. A Fermi smearing of 0.005 Hartree and a real-space cutoff of 4.5 Å were also used to improve the computational performance.

The reactants, transition state, and intermediates generated during ICL formation were modeled in Materials Visualizer and optimized using the conjugated gradient algorithm. The transition state (TS) searches were performed using the complete LST/QST method [56]. In this method, the linear synchronous transit (LST) maximization was performed, followed by energy minimization in directions conjugating to the reaction pathway to obtain an approximated TS. The approximated TS was used to perform quadratic synchronous transit (QST) maximization, and then another conjugated gradient minimization was performed. The cycle was repeated until a stationary point was located. The obtained TS was optimized via eigenvector following, searching for an energy maximum along one previously selected normal mode and a minimum along all other nodes, using the Newton–Raphson method. After this procedure, one transition state was found for each reaction step. Each TS structure was characterized by a vibrational analysis with exactly one imaginary frequency describing the atom motion of the reaction step.

The DNA parameters were analyzed using the online servers Web-3DNA and CURVES+ [40,41]. The values of DNA base-pair step parameters for the native and inter-crosslink structures determined in this study are given in Supplementary Tables S1 and S2.

## 4. Conclusions

Outdoor air pollution is a mixture of multiple pollutants, among which NOx stands out. Due to road traffic, their concentration in the atmosphere has increased in recent decades. Atmospheric pollutants can be sources of DNA damage, as serious as inter-crosslink formation. Starting from the basis that the diazonium intermediate derived from the attack of these compounds is known, we have studied the mechanism of the formation of a crosslink between two guanine nucleobases. We found that the mechanism is thermodynamically favored by a strong stabilization of the formed ICL. In addition, the rate-limiting step process is accessible. In addition, it was found in the MD simulations that the DNA structure is stable with the diazonium intermediate since the loss of one hydrogen bond between the Guanine–Cytosine couple is replaced by cation–π-type interactions. Finally, the presence of the crosslinked guanines forms a nearly planar, covalently linked base pair that does not break the stacking in the DNA structure, aided by the absence of an iminic hydrogen from one of the guanines, resulting in a more significant structural stability of the lesion within the oligonucleotide.

## Data Availability

Not applicable.

## Supplementary Materials

The following supporting information can be downloaded at: https://www.mdpi.com/article/10.3390/ijms231810621/s1. Reference [57] are cited in the supplementary materials.
