# Supplementary material for: Formation Mechanism of Inter-Crosslink in DNA by Nitrogen Oxides Pollutants through A Diazonium Intermediate
Source: Int J Mol Sci. 2022 Sep 13;23(18):10621. doi: 10.3390/ijms231810621 (PMC9502170; doi:10.3390/ijms231810621)
Supplement: Supplementary file 1 [file ijms-23-10621-s001.zip › ijms-1836255-supplementary.pdf]

Table S1. Base Step Parameters from the center of the oligonucleotide in the native molecular dynamics.

	Shift (Å)	Slide (Å)	Rise (Å)	Tilt (°)	Roll (°)	Twist (°)
TC/GA	0.82	0.95	3.12	9.0	2.5	29.5
CC/GG	1.20	1.10	3.77	5.0	6.5	41.0
CG/CG	1.82	0.88	2.99	3.5	9.5	24.5
GG/CC	1.62	0.33	3.77	1.0	3.5	34.0
GA/TC	0.40	0.07	3.26	4.0	4.0	41.0
AT/AT	0.88	0.65	3.40	6.0	5.0	23.5

Table S2. Base Step Parameters from the center of the oligonucleotide in the diazonium molecular dynamics.

	Shift (Å)	Slide (Å)	Rise (Å)	Tilt (°)	Roll (°)	Twist (°)
TC/GA	0.68	1.55	3.69	2.0	7.5	37.0
CC/GG	0.32	1.21	**4.02**	3.0	17.5	35.0
CG/CD	1.13	1.06	3.45	7.5	19.5	16.5
CD/CC	**0.63**	**2.08**	**4.44**	**13.0**	3.0	33.5
GA/TC	1.02	**1.37**	3.19	4.0	4.5	32.0
AT/AT	0.21	2.06	2.76	2.0	2.5	34.0

Statistics Analysis of Molecular Dynamics Data

RMSD. The root mean square deviation of certain atoms in a molecule with respect to a reference structure can be calculated with the program by least-square fitting the structure to the reference structure ($t_2 = 0$) and subsequently calculating the RMSD [57].

$$RMSD(t_1, t_2) = \left[\frac{1}{M} \sum_{i=1}^{N} m_i \left(\boldsymbol{r}_i(t_1) - \boldsymbol{r}_i(t_2) \right)^2 \right]^{1/2}$$

where $M = \sum_{i=1}^{N} m_i$ and $\boldsymbol{r}_i(t)$ is the position of atom i at time t.

RDF. The radial distribution function $g_{AB}(r)$ between particles of type A and B is defined in the following way:

$$g_{AB}(r) = \frac{< \rho_B(r) >}{< \rho_B >_{local}} = \frac{1}{< \rho_B >_{local}} \frac{1}{N_A} \sum_{i \in A}^{N_A} \sum_{j \in B}^{N_B} \frac{\delta(r_{ij} - r)}{4\pi r^2}$$

with $< \rho_B(r) >$ the particle density of type B at a distance r around particles A, and $< \rho_B >_{local}$ the particle density of type B averaged over all spheres around particles A with radius r_{max}. [57].

Propeller Twist Angle. To monitor specific distances between points, the program *gmx distance* can calculate distances as a function of time, as well as the distribution of the distance. The program *gmx angle* calculates the distribution of *angles* and *dihedrals* in time [57].

[57] Apol, E.; Apostolov, R.; Berendsen, H.J.C.; van Buuren, A.; Bjelkmar, P.; van Drunen, R.; Feenstra, A.; Fritsch, S.; Groenhof, G.; Junghans, C.; Hub, J.; Kasson, P.; Kutzner, C.; Lambeth, B.; Larsson, P.; Lemkul, J.A.; Marklund, E.; Meulenhoff, P.; Murtola, T.; Páll, S.; Pronk, S.; Schulz, R.; Shirts, M.; Sijbers, A.; Tieleman, P.; Wennberg, C.; Wolf, M.; Abraham, M.; Hess, B.; van der Spoel, D.; Lindahl, E. Gromacs 2016 Reference Manual.
